# A glycoconjugate of *Haemophilus influenzae* Type *b* capsular polysaccharide with tetanus toxoid protein: hydrodynamic properties mainly influenced by the carbohydrate

**DOI:** 10.1038/srep22208

**Published:** 2016-02-26

**Authors:** Ali Saber Abdelhameed, Gary G. Adams, Gordon A. Morris, Fahad M. Almutairi, Pierre Duvivier, Karel Conrath, Stephen E. Harding

**Affiliations:** 1 National Centre for Macromolecular Hydrodynamics, University of Nottingham, Sutton Bonington LE12 5RD, UK; 2 Department of Pharmaceutical Chemistry, College of Pharmacy, King Saud University, P.O. Box 2457, Riyadh 11451, Saudi Arabia; 3 Insulin and Diabetes Experimental Research (IDER) Group, University of Nottingham, Faculty of Medicine and Health Science, Clifton Boulevard, Nottingham, NG7 2RD UK; 4 Department of Chemical Sciences, School of Applied Science, University of Huddersfield, Queensgate, Huddersfield, HD1 3DH, UK; 5 GSK Vaccines, Rue de l’Institut 89, B-1330 Rixensart, Belgium

## Abstract

Three important physical properties which may affect the performance of glycoconjugate vaccines against serious disease are molar mass (molecular weight), heterogeneity (polydispersity), and conformational flexibility in solution. The dilute solution behaviour of native and activated capsular polyribosylribitol (PRP) polysaccharides extracted from *Haemophilus influenzae* type *b* (*Hib*), and the corresponding glycoconjugate made by conjugating this with the tetanus toxoid (TT) protein have been characterized and compared using a combination of sedimentation equilibrium and sedimentation velocity in the analytical ultracentrifuge with viscometry. The weight average molar mass of the activated material was considerably reduced (M_w_ ~ 0.24 × 10^6^ g.mol^−1^) compared to the native (M_w_ ~ 1.2 × 10^6^ g.mol^−1^). Conjugation with the TT protein yielded large polydisperse structures (of M_w_ ~ 7.4 × 10^6^ g.mol^−1^), but which retained the high degree of flexibility of the native and activated polysaccharide, with frictional ratio, intrinsic viscosity, sedimentation conformation zoning behaviour and persistence length all commensurate with highly flexible coil behaviour and unlike the previously characterised tetanus toxoid protein (slightly extended and hydrodynamically compact structure with an aspect ratio of ~3). This non-protein like behaviour clearly indicates that it is the carbohydrate component which mainly influences the physical behaviour of the glycoconjugate in solution.

*Haemophilus influenzae* is a small non-motile Gram negative bacterium^1^,^2^ present in the nasopharynx of approximately 75 % of healthy children and adults and is regarded as normal flora^3^. A minority (~3–7%) of healthy individuals intermittently harbour the carbohydrate encapsulated *H. influenzae* strains (types *a, b, c, d, e,* and *f* ) in the upper respiratory tract^4^. It was mistakenly thought to be the cause of influenza disease: as a consequence it was named accordingly^5^,^6^. However the considered opinion now is that *H. influenzae* is most likely to have been an important secondary invader to the influenza virus in the 1890 pandemic and many subsequent influenza epidemics. Nonetheless it is still considered responsible for a wide range of clinical diseases such as meningitis and pneumonia^3^,^7^.

Invasive diseases caused by *H. influenzae* seem to occur in humans only and are primarily due to type *b Haemophilus influenzae* or “*Hib*” (>95% of cases) where it remains a major cause of acute bacterial meningitis. Until the availability of the *Hib* vaccine, the type *b H. influenzae* was the main cause of meningitis in children between 6 months and 5 years old, although older children, adolescents and adults can also be infected^8^.

Since 1931 it has been known that some strains of *Haemophilus influenzae* possess a polysaccharide capsule and that there are 6 capsular serotypes (*a–f* )^9^. In 1953, Zamenhof and co-workers^10^ postulated that the type-specific substance of *Haemophilus influenzae* type *b*, was composed of polyribose-ribitol chains with (3→5) phosphate diester linkages between the ribose moieties. Further work^11^,^12^,^13^,^14^ established the double ribose unit as β-D-Ribf-/β-D-Ribf. Anderson and colleagues^15^ and Schneerson and coworkers^16^ have also reported equimolar ratios of phosphorus and ribose. Thus, the polyribosyl ribitol phosphate (PRP) capsule of *Hib* is a linear copolymer composed of repeated units of→3)-*β*-D-Rib*f*-(1→1)-D-ribitol-(5-OPO_3_→[(C_10_H_19_O_12_P)_*n*_] with a defined molecular size^2^,^17^. The size of the polysaccharide used for conjugation should be defined and controlled.

Unlike other bacteria (*e.g Streptococcus pneumoniae* and *Neisseria meningitidis*) the type *b* polysaccharide capsule of *H. influenzae* is attractive as a vaccine antigen since invasive disease is almost exclusively restricted to only one serotype, which in turn has rendered its capsular polysaccharide a prime candidate for vaccine studies. The first generation of *Hib* vaccine was based on the purified type *b* capsular polysaccharide and its ribose-ribitol phosphate repeating units^2^,^18^,^19^,^20^. Conjugation of the polysaccharide with a suitable protein to stimulate a stronger and longer lasting T-cell based immunity^21^ has been the target of research. Connaught Laboratories (now Sanofi Pasteur) produced the first licensed *Hib* conjugate vaccine in 1987 after the pioneering work by Schneerson and co-workers^22^, through random conjugation of PRP to diphtheria toxoid. Following on the heels of this vaccine three other vaccines from the biopharmaceutical companies, namely Wyeth (now Pfizer), Sanofi-Pasteur and Merck, all differing in their carrier protein and method of conjugation, received approval from the Food and Drug Administration (FDA)^2^. Recently approved glycoconjugate vaccines include a *Hib*-Tetanus toxoid (TT) conjugate *Hiberix*^®^ and a novel *Hib*-*MenCY*-TT (*MenHibrix*^®^) by GSK Vaccines. *MenHibrix*^®^ vaccine administered in accordance to the current *Hib* vaccine schedule (USA) would have the potential to induce protective antibodies against *Hib* and meningococcal-CY disease in children^23^.

Important to their function as a vaccine is the structure and stability of the polysaccharide and conjugates thereof. Molecular weight (molar mass) and molar mass distribution have been documented as being the most important physical parameters affecting the immunogenicity of capsular polysaccharides^24^,^25^. While many other aspects of polysaccharide characterisation have been relatively thoroughly explored^26^,^27^,^28^, the physical characterisation of capsular polysaccharides (molecular size and mass distribution and conformational flexibility) has been less extensively pursued. Such physical studies for capsular polysaccharides have been generally limited to low pressure chromatographic analyses calibrated with “standards” and more recently to high performance size exclusion chromatography (SEC) coupled to on-line refractive index detector (RI), multi-angle light scattering (MALS). In this study we used the analytical ultracentrifuge due to its larger dynamic range (molar masses from 10^3^ to >10^8^ g.mol^−1^), particularly appropriate for large glycoconjugates, many of which are beyond the exclusion limit of SEC-MALS^29^. This study is designed principally to characterise the purified native and activated capsular polyribosylribitol polysaccharides (PRP) from *Haemophilus influenzae* type *b* (referred to as PRP native and PRP-ADH respectively) as well as the final PRP*-* TT conjugate (with a polysaccharide: protein ratio of ca. 0.4) and to establish whether it is the protein component or carbohydrate component which principally influences the physical or hydrodynamic properties in solution This study has also been designed to demonstrate the usefulness of analytical ultracentrifuge based procedures – all not requiring a separation column or matrix - in the characterisation of large glycoconjugate vaccines.

## Results

**Figure 1:**
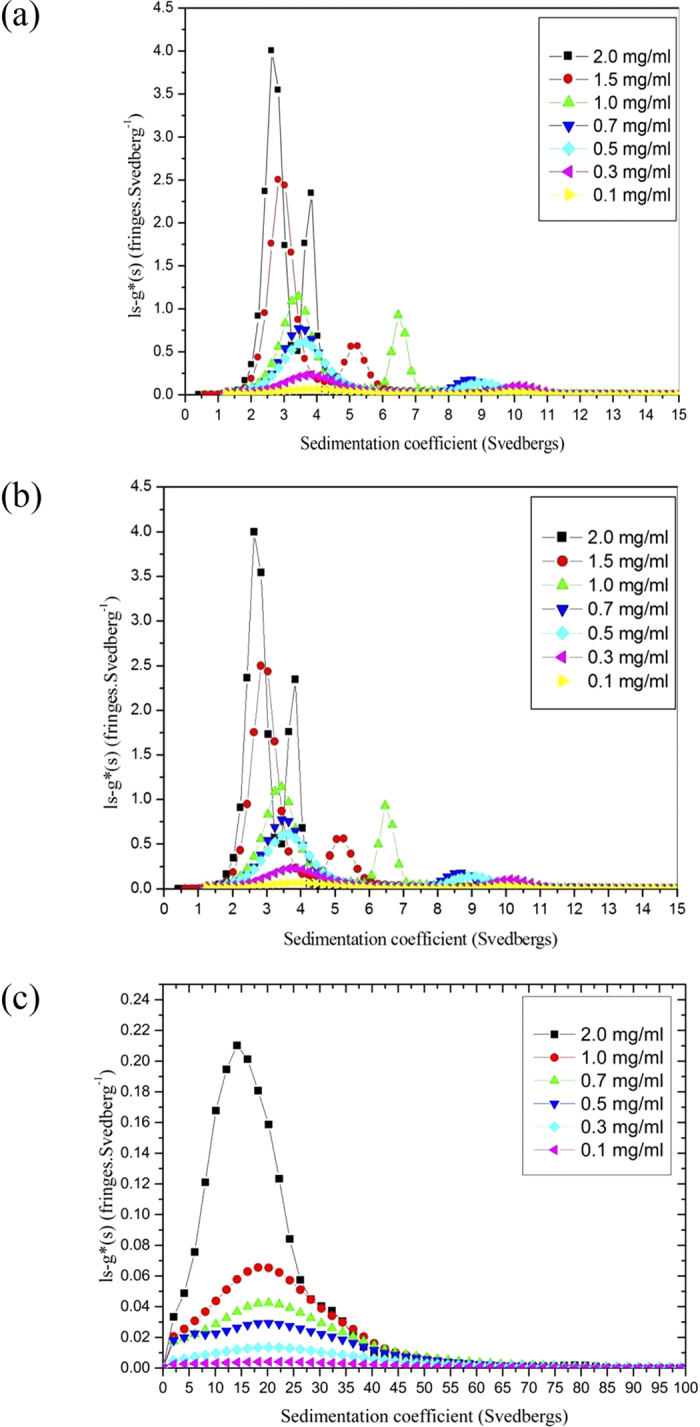
Sedimentation coefficient distributions, g*(*s*) vs *s* profiles, at different concentrations for (**a**) *Hib* PRP-native capsular polysaccharide (**b**) *Hib* PRP-ADG (**c**) *Hib* PRP-TT conjugate. The apparent sharpening of the peaks as the concentration increases is due to “hypersharpening” through the combined effects of polydispersity and non-ideality: the faster moving species in a distribution are slowed down by having to sediment through a solution of the slower ones. These effects diminish as the concentration is reduced.

**Figure 2:**
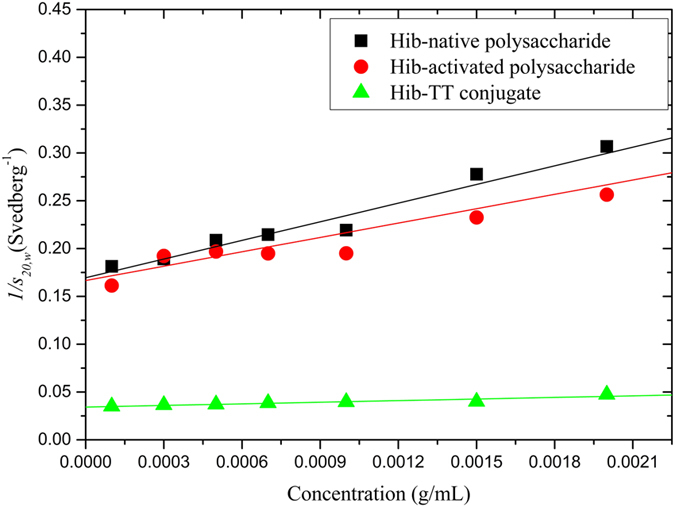
Concentration dependence (reciprocal) sedimentation coefficient plot for *Hib* PRP-native, *Hib* PRP-ADH and *Hib* PRP-TT, to remove the effects on non-ideality. Sedimentation coefficients measured in the phosphate chloride buffer (pH = 6.8, I = 0.10) had been normalized to standard conditions (the viscosity and density of water at a temperature of 20.0 °C).

Sedimentation velocity in the analytical ultracentrifuge was first applied as the primary method for assessing the size heterogeneity. Bimodal plots of apparent sedimentation coefficient distributions g*(*s*) versus *s* were seen in the case of both native *Hib* polysaccharide (Fig. 1a) and the ADH activated *Hib* (Fig. 1b) whereas a unimodal profile for the much larger conjugate structure was observed (Fig. 1c). This does not mean that the conjugate was monodisperse as the broadness of the peak is commensurate with a broad distribution of sizes of a (quasi-) continuous type arising from polydispersity of the carbohydrate chains. Under these conditions *Hib* polysaccharides and conjugate have apparent weight average sedimentation coefficients ranging from 5.9S to 30S as reported in Table 1. All three showed classical dependencies of *s*_*20,w*_ on *c*, (decrease of *s*_*20,w*_ with increase of *c*) indicative of non-ideality and the absence of significant reversible associative effects (Fig. 2).

**Figure 3:**
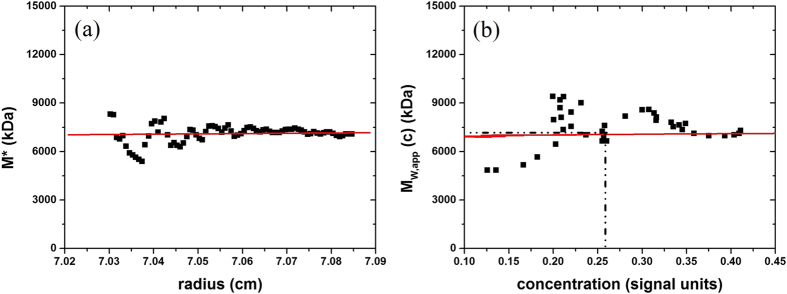
*SEDFIT-MSTAR* output for analysis of *Hib* PRP-TT conjugate at a loading concentration of 0.3 mg.mL^−1^ to find the (apparent) weight average molar mass *M*_w,app_ over the whole distribution. (**a**) The operational point average molar mass M*(*r*) plotted as a function of radial position from the centre of rotation *r*. *M*_w,app_, the (apparent) weight average molar mass for the whole distribution being measured = M* extrapolated to the radial position at the cell base. Retrieved *M*_w,app_ from this extrapolation = (7.3 ± 0.4) × 10^6^ g.mol^−1^. (**b**) Plot of the “point” or “local” apparent average molar mass *M*_w,app_(*r*) at radial positions *r*, as a function of local concentration *c*(*r*) in the ultracentrifuge cell. The “hinge point” corresponds to the radial position where the *c*(*r*) = the initial loading concentration. At this hinge point *M*_w,app_(*r*) = (7.3 ± 0.5) × 10^6^ g.mol^−1^. Although not as precise a way of estimating *M*_w,app_ from the sedimentation equilibrium records it does provide an internal check for consistency.

Molar mass values estimated from sedimentation equilibrium using the *SEDFIT-MSTAR* and *MFIT* algorithms for weight-average molar mass and z-average molar mass, respectively are shown in Table 1 with an example of a determination for the PRP-TT conjugate in Fig. 3. The weight average molar mass values for the glycoconjugate obtained using the matrix-free technique of sedimentation in the analytical ultracentrifuge^29^ appear to be commensurate with a recent study by Lockyer and coworkers^30^ using size exclusion chromatography (SEC) coupled to multi-angle light scattering, but without the problem of large molar mass species (>2 × 10^6^) eluting in the void volume of the SEC.

**Figure 4:**
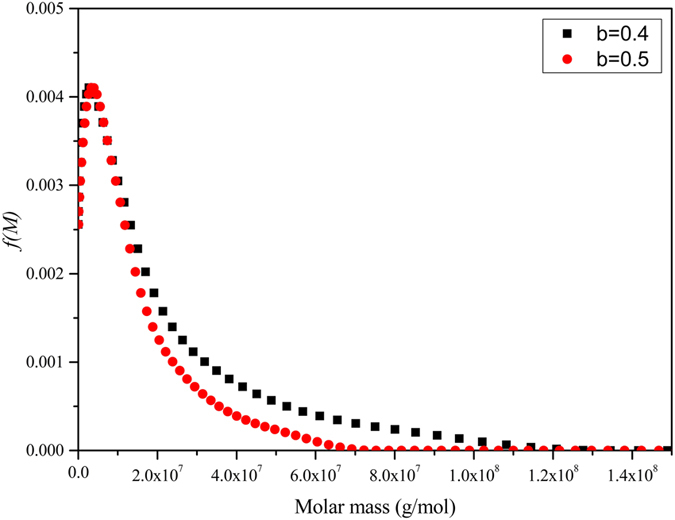
Molar mass distribution *f(M)* profile from sedimentation velocity for *Hib* PRP-TT conjugate. Obtained by transforming the sedimentation coefficient distribution of Fig. 1c by the Extended Fujita method, using the weight average sedimentation coefficient with the weight average *M*_w,app_ (from Fig. 3) molar mass and two different plausible values of the conformation parameter *b*. The broad distribution is completely different for the sharp monomer-dimer distribution we observed earlier for TT by itself 33.

The sedimentation coefficient distribution – which itself gives a good measure of sample heterogeneity - for *Hib* PRP-TT conjugate was then transformed into a corresponding distribution of molar mass using the *Extended Fujita Approach* of Harding *et al.*^31^ (Fig. 4).

The transformation is as follows:


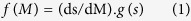


with


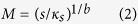


and


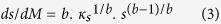


*b* is a conformation parameter that has already been estimated for a number of polysaccharides^32^ and κ_s_ can be found from equation (3) provided that at least one value of *M* (*e.g. M*_w_ from sedimentation equilibrium) is known for one value of *s* (*e.g.* the weight average *s* value). The distributions so obtained for two plausible values of *b* are shown in Fig. 4. The broad distribution is completely different for the sharp monomer-dimer distribution we observed earlier for TT by itself 33.

For macromolecules of known molar mass the intrinsic viscosity [η] can provide an important measure of the conformational flexibility (especially when used in conjunction with the sedimentation coefficient, *s*^o^_20,w_, and the concentration dependence coefficient *k*_s_). Three different and complementary extrapolation methods values to zero concentration (to eliminate complications through non-ideality effects) were used for all 3 samples – all gave good agreement and are shown in Table 1.

## Discussion

The hydrodynamic data collectively enable us to establish what is the conformational flexibility of the *Hib* glycoconjugate and to establish whether it is either the protein component or the carbohydrate component which is more strongly influencing the hydrodynamic properties. There are four approaches, one using the sedimentation data alone, the others using various combinations.

### Conformational analysis: translational frictional ratio

The translational frictional ratio, *f/f*_*o*_ is a parameter which depends on conformation *and* molecular expansion through hydration effects^34^. It can be measured experimentally from the sedimentation coefficient and molar mass:


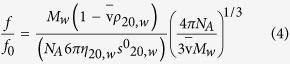


where N_A_ is Avogadro’s number. *f* is the friction coefficient of the molecule and *f*_*o*_ the corresponding value for a spherical particle of the same mass and (anhydrous) volume^31^. Departures from *f/f*_*o*_ = 1 are due to either asymmetry (for example the modest asymmetries seen in the tetanus toxoid protein – Table 1 and ref. 33) or an increase in volume due to swelling through particle solvation effects – the high values in Table 1 could be due to either (or both) and without other information it is impossible to distinguish between the two. Combination with viscosity data and how viscosity and the sedimentation coefficient change with molar mas, however, help us to be more specific.

### Conformational analysis: the Wales-van Holde ratio

The Wales-van Holde ratio^35^, *R* = *k*_*s*_*/[η]* - where *k*_*s*_ is the concentration dependence of the sedimentation coefficient or “Gralen” coefficient - is perhaps the simplest indicator of a macromolecules conformational flexibility in solution. The limits are ~1.6 for a compact sphere or a non-draining random coil, and ~0.1 for a stiff rod^36^. From Table 1 it seems that the values for the polysaccharide and glycoconjugate are identical (R ~ 0.9) and consistent with a flexible coil structure.

### Conformational analysis: Estimation of the Persistence Length *L*_p_

For a more quantitative estimate of chain flexibility we can use the persistence length *L*_*p*_, which has theoretical limits of 0 for a random coil and ∞ for a stiff rod. Practically the limits are ∼1–2 nm for a random coil (such as the polysaccharide pullulan) and ∼200–300 nm for a very stiff rod shaped macromolecule (such as xanthan or schizophyllan)^29^. Several methods are available for the estimation of *L*_*p*_ using either intrinsic viscosity^37^,^38^,^39^ or sedimentation coefficient^40^ measurements. For example the Bohdanecky-Bushin relation


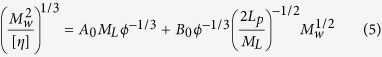


where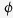 is the Flory-Fox coefficient (2.86 × 10^23^ mol^−1^) and A_0_ and B_0_ are tabulated coefficients, and the Yamakawa–Fujii equation^40^


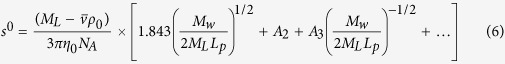


**Figure 5:**
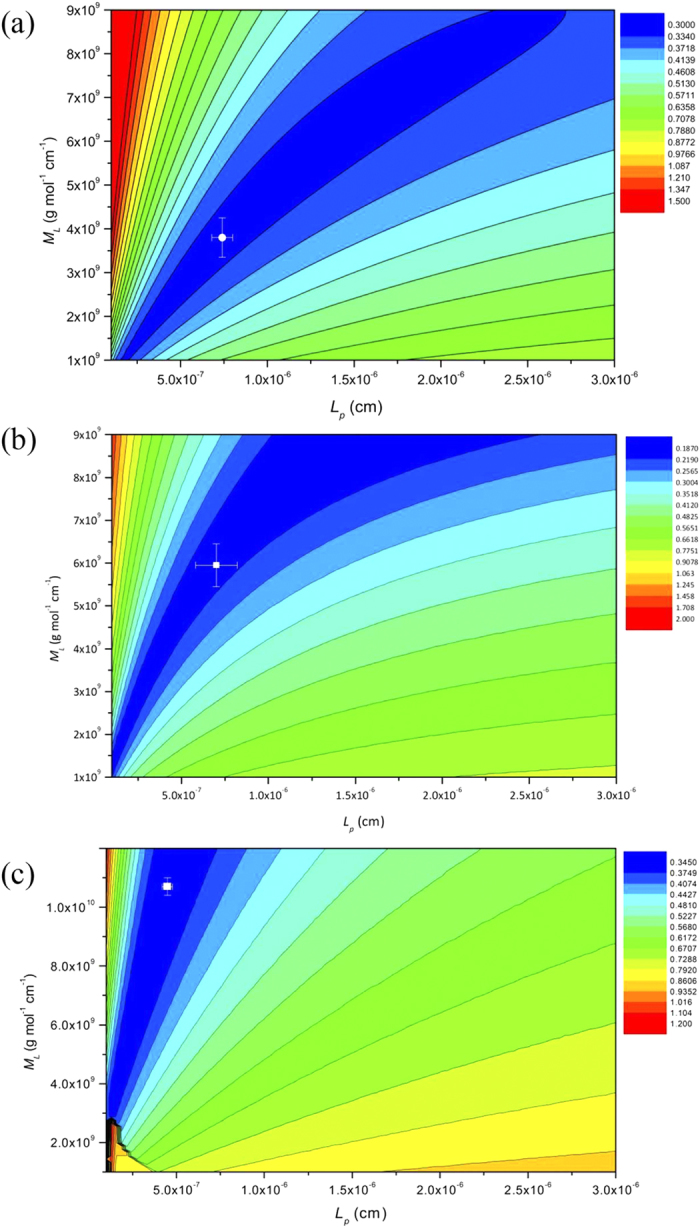
Plot of mass per unit length *M*_*L*_ versus persistence length *L*_*p*_ evaluation using the multi-HYDFIT procedure of Ortega and Garcia de la Torre^41^. (**a**) the *Hib* PRP-native polysaccharide. The plot yields *L*_*p*_ ∼ 7.0 × 10^−7^ (cm) and *M*_*L*_ ∼ 3.8 × 10^9^ (g.mol^−1^.cm^−1^) at the minimum target (error) function (indicated by the white cross). (**b**) Hib PRP-ADH, *L*_*p*_ ∼ 7.0 × 10^−7^ (cm) and *M*_*L *_∼ 6.0 × 10^9^ (g. mol^−1^. cm^−1^); (**c**) Hib PRP-TT *L*_*p*_ ∼ 4.5 × 10^−7^ (cm) and *M*_*L*_ ∼ 10.7 × 10^9^ (g. mol^−1^. cm^−1^).

Yamakawa and Fujii^39^ showed that A_2_ can be considered as –*ln(d/2L*_*p*_) and A_3_ = 0.1382 if the *L*_*p*_ is much higher than the chain diameter, *d*. Difficulties arise if the mass per unit length is not known, although both relations have recently been built into an algorithm Multi-HYDFIT^40^ which estimates the best estimates or best range of values of *L*_*p*_ and *M*_*L*_ based on minimization of a target function *Δ*. An estimate for the chain diameter *d* is also required but extensive simulations have shown that the results returned for *L*_*p*_ are relatively insensitive to the value chosen for *d* which was fixed at an average of ∼0.8 nm^41^,^42^. *M*_*L*_ and *L*_*p*_ were treated as variables and the minimum value of the target function *Δ* was estimated on a 2D contour plot for each sample (see Fig. 5) and the values estimated given in Table 2. All the values are consistent with flexible random coil structures with persistence lengths between 4.5 and 7 nm.

### Sedimentation Conformation Zoning

**Figure 6:**
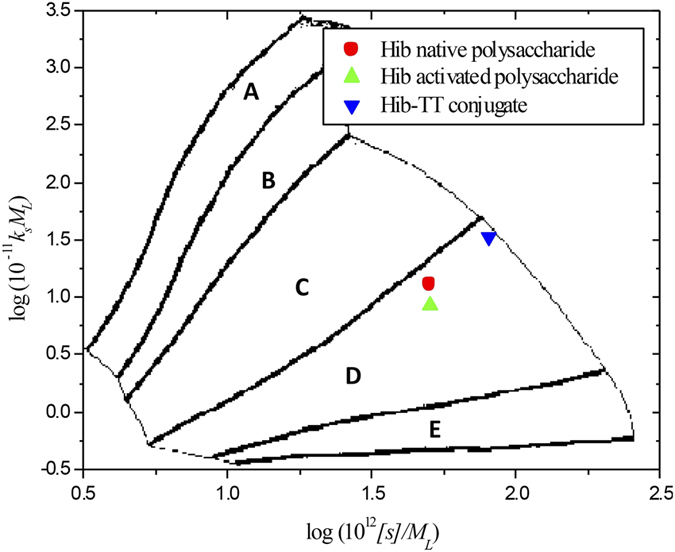
Conformation Zoning plot, *Hib* PRP*-*native, PRP-ADH and PRP-TT conjugate (with spacer) all have very flexible structures in the “Zone D” region close to Zone C Zone A: Rigid rod with no flexibility; Zone B: Rigid rod with some flexibility; Zone C: Semi-flexible coil; Zone D: Random coil; Zone E: Globular or heavily branched structures. See ref. 56.

This high flexibility is confirmed by “Sedimentation Conformation Zoning”, introduced by Pavlov *et al.*^43^,^44^. This involves plotting *k*_*s*_*M*_*L*_ versus [*s*]/*M*_*L,*_onto a zonal template established from macromolecules of known conformation type. *k*_*s*_ is the Gralen coefficient, *M*_*L*_ the mass per unit length and the “intrinsic” sedimentation coefficient [*s*] is given by:


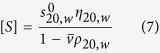


with η_20,w_ and ρ_20,w_ the viscosity and density of water at 20.0 °C, and 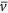 the partial specific volume. Figure 6 – confirms that both the native and activated polysaccharides, and the glycoconjugate are all highly flexible structures (falling just within the Zone D area – randomly coiled structures), and not too different with capsular polysaccharides from *Streptococcus pneumoniae*^45^ (Zone C – semi-flexible). The tetanus toxoid protein by itself - slightly extended and hydrodynamically compact^34^ – is by contrast a “Zone E” particle.

All four conformation approaches (frictional ratio, Wales-van Holde ratio, persistence length and conformation zoning) show that conjugation with the tetanus protein yielded large polydisperse structures of (M_w_ ~ 7.3 × 10^6^ g/mol), but which retained the high flexibility of the native and activated polysaccharide, similar to what we found earlier for capsular polysaccharides from *Streptococcus pneumoniae*^45^ – and very different for the previously characterised tetanus toxoid protein^34^, as summarized in Table 1. These findings supplement other biophysical studies on other glycoconjugate vaccines. The high flexibility arising from carbohydrate chains is consistent for example with the ^1^H-NMR relaxation studies of Berti and coworkers^46^ on *Hib* conjugated to the non-toxic mutant of diphtheria toxin CRM197: this work also provided evidence of reduced hydration of the protein as a result of the conjugation. Using circular dichroism and fluorescence spectroscopy and ^1^H-NMR, the protein component of *Hib* and *MenC* glycoconjugate vaccines was nonetheless found to be important for pH and thermal stability^47^,^48^. In more recent work Pecetta^49^ and coworkers have provided evidence based on differential scanning calorimetry and circular dichroism of conjugation causing some structural changes of the CRM197 protein.

### Concluding Remarks

The flexible chain-like hydrodynamic properties of the PRP-TT glycoconjugates are commensurate with those of a polysaccharide rather than a protein. Tetanus toxoid protein is known to be a globular protein of aspect ratio ~3:1 with a tendancy to form small amounts of dimer (~14%)^34^. Intrinsic viscosities of the protein are small and typical of globular proteins and very different from the *Hib* polysaccharide and glycoconjugate. This clearly indicates that it is the carbohydrate component which most strongly influences the physical behaviour of these substances in solution. This study has also shown the usefulness of the analytical ultracentrifuge as a matrix free method for ‘quality control’ assessment of the heterogeneity and conformational flexibility of the *Hib* vaccine preparations.

## Methods

### Sample preparation

The bulk *Hib* manufacturing process consists of the following steps: after initial solid preculture steps, the bacteria are incubated in shake flasks. Once the appropriate cell density is reached, the inoculum is transferred to a fermentor for further amplification. The virulent strain of Hib is grown in a medium supplemented with hematin and nicotinamide adenine dinucleotide (NAD). During the growth phase, the pH is regulated with concentrated NaOH solution and the dissolved oxygen is regulated by the stirring speed and air flow rate. At the onset of the drift phase (7–8 hours after inoculation), the pH and dissolved oxygen regulations are switched off and the parameters are left to drift. The fermentation is terminated 10 hours after the inoculation. The broth containing the polyribosyl (PRP) polysaccharide of interest is inactivated by heat and centrifuged to remove cell debris. Purification is assured by *Hib* precipitation with Cetavlon in the presence of Celite. The complex PRP-CTAB is an insoluble complex retained on the Celite. The PRP polysaccharide is detached from the Cetavlon by using a high salt concentration (0.5 M NaCl). The product is ultrafiltered to eliminate residual nucleic acids, then precipitated with ethanol, dried and kept at −20 °C until conjugation. The residuals (DNA (<0.2%), proteins (<0.1%), LPS(<0.03%)) were very low in the final bulk of polysaccharide and met all the required specifications of the pharmacopeia (WHO/EMEA).

*Hib* PRP-polysaccharide activation (using adipic acid dihydrazide as linker) prior to its conjugation was using the cyanogen bromide method^50^,^51^,^52^. By-products were removed by further ultrafiltration (30 kDa cut-off). The conjugation process did not involve oxidization of PRP with periodic acid: the PRP is directly activated. After activation (6 minutes), the ADH is added to form a covalent polysaccharide-ADH product which is then able to be conjugated with the –COOH groups of the tetanus toxoid (TT) protein by using carbodimide conjugation. The conjugation between the activated (PRP-ADH) and the TT protein was performed using EDC (1-ethyl-3-(3-dimethylaminopropyl)-carbodimide hydrochloride)^53^,^54^. The *Hib PRPTT* conjugate was then purified on a Sephacryl S500HR column using 0.2 M NaCl as the elution solution. The conjugate was then sterile filtered before a final ultrafiltration step, which led to the product meeting the required specifications of the pharmacopeia, with <15% of unconjugated polysaccharide and <5% unconjugated protein. All samples were dissolved in phosphate buffered saline pH ~6.8, I = 0.1 M^55^, at 20.0 °C and mixed by magnetic stirring at room temperature for 24 hours. All solutions were then diluted to the appropriate (total macromolecular) concentrations required.

### Sedimentation velocity in the analytical ultracentrifuge

A general description of the ultracentrifugal methods and how they can be used for glycoconjugate and polysaccharide characterisation is given in two recent references^29^,^56^. Specifically for this study sedimentation velocity experiments were performed using a Beckman (Palo Alto, CA, USA) Optima XL-I analytical ultracentrifuge equipped with Rayleigh interference optics and an automatic on-line data capture system. Conventional 12 mm double-sector epoxy cells with sapphire windows were loaded with 400 μL of different (total macromolecular) concentrations (0.1–2.0 mg mL^−1^) of each sample and a matching amount of the corresponding reference buffer (phosphate buffered saline) in appropriate channels. Samples were run at a rotor speed of 45000 rpm (~150,000 g) at a temperature of 20.0 °C. Concentration profiles and the movement of the sedimenting boundary in the analytical ultracentrifuge cell were recorded using the Rayleigh interference optical system and converted to concentration (in units of fringe displacement relative to the meniscus, *j*) versus radial position, *r*^26^. Data was analysed using the least squares boundary ls-g*(s) model incorporated into the SEDFIT analytical algorithm of Dam & Schuck^57^. SEDFIT generates an apparent distribution of sedimentation coefficients in the form of g*(s) versus *s*, where *s* is the sedimentation coefficient (in Svedberg units S = 10^−13^ sec). The * indicates the profiles are not corrected for diffusion broadening (likely to be small for slow-diffusing polysaccharides and glycoconjugates). This data analysis was followed by the correction to standard solvent conditions - namely the density and viscosity of water at 20.0 °C - to yield *s*_*20,W*_ using the algorithm SEDNTERP^58^, which also incorporates the partial specific volume (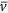) of the samples 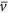 ~ 0.63 mL.g^−1^. To account for hydrodynamic non-ideality (co-exclusion and backflow effects), the apparent sedimentation coefficients (*s*_*20,w*_) were calculated at a series of different cell loading concentration and extrapolated to infinite dilution using the Gralén relation^59^:


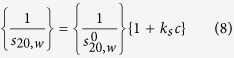


where *k*_*s*_ is the Gralén or concentration dependence coefficient.

### Sedimentation equilibrium in the analytical ultracentrifuge

Sedimentation equilibrium experiments were also performed using the Beckman (Palo Alto, CA, USA) Optima XL-I analytical ultracentrifuge again using the Rayleigh interference optics and an automatic on-line data capture system to record equilibrium concentration distribution profiles. The modified long (20 mm) optical path length double-sector titanium cells with sapphire windows were selected and loaded with 0.070 mL of solution (dialysed at room temperature against the phosphate buffered saline for 48 hours) and a matching amount of reference buffer dialysate in the appropriate channels. Samples were centrifuged at rotor speeds selected to give a sufficient fringe increment from meniscus to base^60^ *i.e.* 4000 rpm (~1200 g), 9000 rpm (~5900 g) and 2000 rpm (~300 g) for PRP native, PRP-ADH and the PRP-TT solution, respectively, at a temperature of 20.0 °C. Scans were taken every one hour and equilibrium was reached after approximately 48–72 hours. Optical records (Rayleigh interference profiles) of the relative concentration distribution of the solute at equilibrium were analysed to give the weight (mass) average apparent molar mass *M*_*w,app*_ using the *SEDFIT-MSTAR* algorithm^61^. This uses the M* function of Creeth and Harding^62^, together with the hinge point method (evaluation of the point or weight average molar mass at the radial position in the distribution where the local (total macromolecule) concentration *c(r)* = the initial loading concentration, *c*^61^). The use of long path length cells meant that low loading concentrations could be used to give a sufficient signal (~0.3 mg.mL^−1^). At such low concentrations, non-ideality effects (which tend to lead to underestimates of the molar mass) may be relatively small and we make the approximation that the apparent weight average molar mass *M*_*w,app*_ is equal to the true weight average molar mass *M*_*w*_^60^. We also estimate the apparent z-average molar mass *M*_*z,app*_ using the *MFIT* algorithm of Ang and Rowe^63^.

### Viscometry

Dynamic viscosity measurements for *Hib* native, *Hib*-ADH and *Hib*-TT, were carried out using the automated micro-viscometer Anton Parr AMVn (Anton Parr, Graz, Austria) at a concentration series (total macromolecular concentration) from 0.1–2.0 mg mL^−1^ based on the rolling ball viscosity method in which the apparatus measures the time of a (silanized) steel ball needed to roll in a 1.6 mm diameter silanized glass capillary containing the sample. The experiment was performed at different reclining angles of 70° *n* = 4 times), 60° (*n* = 4 times) and 50° (*n* = 6 times) under precise temperature control (20.00 ± 0.01 °C). Huggins^64^ and Kraemer^65^ extrapolations forms (see, also ref. 38) were performed to obtain the intrinsic viscosity (Equations 9, 10). Intrinsic viscosities were also estimated using the Solomon – Ciutâ relation (Equation 11)^66^.


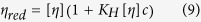



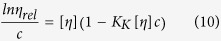



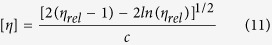


where *c* is the concentration, 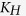 and 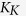
are the Huggins and Kraemer coefficients, repectively.

## Additional Information

**How to cite this article**: Abdelhameed, A. S. *et al.* A glycoconjugate of *Haemophilus influenzae* Type *b* capsular polysaccharide with tetanus toxoid protein: hydrodynamic properties mainly influenced by the carbohydrate. *Sci. Rep.* **6**, 22208; doi: 10.1038/srep22208 (2016).

## Figures and Tables

**Table 1:** Hydrodynamic properties for *Hib* PRP-native, *Hib* PRP-ADH and *Hib* PRP-TT derivatives.

Sample	*s*^*o*^_*20,w*_ (S)	*k*_*s*_ mL.g^−1^	10^−3^ × *M*_*w*_^a^ g.mol^−1^	10^−3^ × *M*_*z*_^b^ g.mol^−1^	[η]^c^ mL.g^−1^	[η]^d^ mL.g^−1^	[η]^e^ mL.g^−1^	*k*_s_*/*[η]	*f/f*_*o*_
PRP-native	5.9 ± 0.2	400 ± 40	1200 ± 50	1250 ± 60	447 ± 14	445 ± 8	445 ± 6	0.9 ± 1	9.8
PRP-ADH	6.0 ± 0.2	260 ± 40	240 ± 10	325 ± 20	275 ± 4	273 ± 3	275 ± 3	0.9 ± 2	3.3
PRP-TT	30.0 ± 0.5	190 ± 10	7300 ± 420	7700 ± 390	225 ± 2	224 ± 6	224 ± 4	0.9 ± 1	6.4
*TT*^f^ *monomer*	7.6 ± 0.1		150 ± 5		5.7 ± 0.1	5.7 ± 0.1			1.3
*TT*^f^ *dimer*	11.6 ± 0.2		270 ± 15						

In phosphate-chloride buffer (pH = 6.8, I = 0.10).

^a^Sedimentation equilibrium SEDFIT-MSTAR analysis.

^b^Sedimentation equilibrium MFIT analysis.

^c^Huggins extrapolation procedure.

^d^Kraemer extrapolation procedure.

^e^Solomon-Ciuta procedure.

^f^from ref. 33.

**Table 2:** Values for the mass per unit length *M*_*L*_ and the persistence length *L*_*p*_ from global hydrodynamic analysis for PRP-native, PRP-ADH and PRP-TT.

Sample	10^−7^ × *M*_*L*_ (g.mol^−1^.cm^−1^)	10^7^ × *L*_*p*_ (cm)
PRP-native	380 ± 50	7.0 ± 1.0
PRP-ADH	600 ± 50	7.0 ± 1.2
PRP-TT	1070 ± 50	4.5 ± 0.3
